# Society for Cardiovascular Magnetic Resonance President's page

**DOI:** 10.1186/1532-429X-11-32

**Published:** 2009-08-19

**Authors:** Christopher M Kramer

**Affiliations:** 1 University of Virginia Health System, Departments of Medicine and Radiology, Lee Street, Box 800170, Charlottesville, VA 22908, USA

## Abstract

The year 2009 has been a busy one for the SCMR. With health care reform in the headlines, SCMR is committed to expanding the use of CMR in clinical practice and research. Progress is being made quickly as the U.S. moves towards approval of payment for flow and in Europe, publication of the CMR registry is nigh. The impact factor of JCMR jumped up and the program for the 2010 meeting in Phoenix looks outstanding. All in all, SCMR is making advancements in many fronts.

## Review

I write this report as many of you have been, are presently on, or are about to take your summer vacation/holidays. I trust that they were safe and restful. The summer has been anything but restful for SCMR and your leadership team. It has been both exciting and challenging as new opportunities have arisen. The SCMR Board of Trustees had its annual summer meeting, this year at the London Heathrow Hilton. The agenda was quite full and I am happy to report that the future continues to look bright for SCMR.

In regards to the SCMR '10 Annual Scientific Session, Program Chair Andrew Arai MD and Abstract Chair Sven Plein MD have a terrific program in store. Highlights will include an opening plenary on CMR and outcomes research, past, present, and future, and a closing plenary on the 5 best clinical indications for CMR. Three parallel tracks are planned for clinicians, basic scientists, and pediatric imagers. Both basic science/high field and introductory physician pre-courses will be held on the day prior to the start of the regular sessions. We suspect this will be one of the best annual meetings to date. Please remember the abstract deadline of September 15^th ^and make your plans to come to warm, sunny Phoenix, Arizona from January 21–24, 2010.

We continue to work closely with the nucleus of the European Working Group of CMR to finalize plans for the February 2011 meeting in Nice, France. In addition, SCMR is working with the European Working Group on a rewrite of the 2004 consensus paper on Clinical Indications for CMR. The 2012 meeting will likely be in Orlando, Florida, and the location of the 2013 meeting remains to be finalized, although San Francisco is the likely venue. Thus, an outstanding series of locales are planned for upcoming annual meetings.

Great news was recently heard by Professor Dudley Pennell, editor of *the Journal of Cardiovascular Magnetic Resonance*, on the increase in the journal's impact factor from 1.87 to 2.15. This jump of 15% reflects both his and previous editor Gerry Pohost MD's tireless efforts on behalf of the journal. It is very much appreciated by SCMR's leadership and the membership. Please continue to send your best scientific work to our flagship journal.

Meanwhile, the SCMR website has migrated to its new host and now can be updated in real time by the website committee and SCMR headquarters. Kudos should be given to James Moon and the website committee for making the website a user friendly endeavor.

On the regional chapter front, steady progress is being made. The European Chapter has made great headway with its CMR registry and the first report on its successes is due out shortly in the *Journal of the American College of Cardiology*. A Middle Eastern Chapter is in the planning stages. Through all the hubbub surrounding health care reform in the U.S., the U.S. Chapter led by Scott Flamm MD has successfully worked with the American College of Radiology (ACR) and the American College of Cardiology (ACC) to persuade the Center for Medicare Services (CMS) to revisit its national coverage determination to not cover blood flow measurement by CMR. CMS is now considering allowing local Medicare carriers to make coverage determinations on CMR blood flow measurement at their own discretion. It is hoped that these changes will be instituted by January 1, 2010. This is great news for both patients and CMR practitioners as this important part of a complete CMR examination should now be covered. Meanwhile, multi-modality appropriate use criteria for imaging in heart failure are under development by the ACC and ACR in partnership. CMR will play a significant role. The next document planned is imaging of patients with chest pain in the emergency department. Further documents will be rolled out in an ongoing manner.

Exciting news continue to emanate from the Clinical Trials Committee. The SCMR-AMI (Superior Classification by Magnetic Resonance of Acute Myocardial Infarction) study is well into the planning stages. Eike Nagel MD, Jeanette Shulz-Menger MD, and I are on the Executive Steering Committee for this 3000 patient, 5-year planned study comparing CMR to echocardiography in the prediction of death and other cardiovascular outcomes. A letter was sent to Michael Lauer, Director of the Division of Cardiovascular Sciences at the NHLBI in late July asking permission to submit the full proposal for funding consideration. Should it be funded, many of your CMR sites will be considered as enrollment sites. The data coordinating center for the study will be the Christiana Care Center for Outcomes Research led by William Weintraub MD and the clinical coordinating center will be ICON Clinical Research. Matthias Friedrich MD will run the CMR core lab and Scott Solomon MD will run the echocardiographic core lab out of the Brigham and Women's Hospital. Consultants include Leslee Shaw PhD for economic outcome analysis and John Spertus MD for health outcomes analysis. We are certainly hopeful that NHLBI will look favorably on this study in the era of comparative effectiveness research.

In other news, SCMR's financial outlook and membership remain in very positive territory. Strategies are being put in place to aim to increase memberships in traditionally underrepresented countries such as China and others in Asia. Vice President Eike Nagel MD is putting together a committee to update the 2007 document on training and credentialing in CMR. New guidelines for technologist credentialing were compiled by Mercedes Pereyra and the technologist committee and were approved by the board. These should go far towards standardizing what is expected for technologists who perform CMR. Board members are examining a potential partnering with the ACC on a educational course in the spring as well as rewriting the CMRSAP disk/online tutorial that was last updated in 2005.

In summary, it has been a very active 6 months for SCMR. Despite difficult financial times world-wide and healthcare crises in the U.S. and elsewhere, your society remains robust and forward-looking. Credit is due to your Board of Trustees, SCMR committees, Deborah Berkowitz and the team at Talley Management, and you, the membership. We will plan to see you in Phoenix!

## Competing interests

The author declares that they have no competing interests.

